# cDNA microarray analysis of the effect of cantharidin on DNA damage, cell cycle and apoptosis-associated gene expression in NCI-H460 human lung cancer cells *in vitro*

**DOI:** 10.3892/mmr.2015.3538

**Published:** 2015-03-24

**Authors:** TE-CHUN HSIA, CHIEN-CHIH YU, SHU-CHUN HSU, NOU-YING TANG, HSU-FENG LU, CHUN-SHU YU, SHIN-HWAR WU, JAUNG-GENG LIN, JING-GUNG CHUNG

**Affiliations:** 1 Graduate Institute of Chinese Medicine, China Medical University, Taichung 40402, Taiwan, R.O.C.; 2 Department of Internal Medicine, China Medical University Hospital, Taichung 40402, Taiwan, R.O.C.; 3 School of Pharmacy, China Medical University, Taichung 40402, Taiwan, R.O.C.; 4 Department of Biological Science and Technology, China Medical University, Taichung 40402, Taiwan, R.O.C.; 5 Department of Clinical Pathology, Cheng Hsin General Hospital, Taipei 11220, Taiwan, R.O.C.; 6 Division of Critical Care Medicine, Department of Medicine, Changhua Christian Hospital, Changhua 50006, Taiwan, R.O.C.; 7 Department of Biotechnology, Asia University, Taichung 41354, Taiwan, R.O.C.

**Keywords:** cantharidin, H460 cells, DNA damage, cell cycle, apoptosis, *in vitro*

## Abstract

Cantharidin (CTD) induces cytotoxic effects in different types of human cancer cell; however, to date, there have been no studies on the effects of CTD on gene expression in human lung cancer cells and the potential associated signaling pathways. Therefore, the present study aimed to investigate how CTD affects the expression of key genes and functional pathways of human H460 lung cancer cells using complementary DNA microarray analysis. Human H460 lung cancer cells were cultured for 24 h in the presence or absence of 10 *μ*M CTD; gene expression was then examined using microarray analysis. The results indicated that 8 genes were upregulated > 4-fold, 29 genes were upregulated >3–4-fold and 156 genes were upregulated >2–3-fold. In addition, 1 gene was downregulated >4 fold, 14 genes were downregulated >3–4-fold and 150 genes were downregulated >2–3 fold in H460 cells following exposure to CTD. It was found that CTD affected DNA damage genes, including DNIT3 and GADD45A, which were upregulated 2.26- and 2.60-fold, respectively, as well as DdiT4, which was downregulated 3.14-fold. In addition, the expression of genes associated with the cell cycle progression were altered, including CCND2, CDKL3 and RASA4, which were upregulated 2.72-, 2.19- and 2.72-fold, respectively; however, CDC42EP3 was downregulated 2.16-fold. Furthermore, apoptosis-associated genes were differentially expressed, including CARD6, which was upregulated 3.54-fold. In conclusion, the present study demonstrated that CTD affected the expression of genes associated with DNA damage, cell cycle progression and apoptotic cell death in human lung cancer H460 cells.

## Introduction

Lung cancer accounts for ~28% of cancer-associated mortali-ties (1), the occurrence of which is increasing worldwide. There are ~1.2 million novel cases of lung cancer and ~1 million mortalities from lung cancer each year (2). Lung cancer may be subdivided into small cell lung carcinoma and non-small cell lung carcinoma (NSCLC). The majority of lung cancer diagnoses are NSCLC (3,4), which has a five-year survival rate of ~33% (5). At present, the standard treatment for patients with resectable stage I to IIIA NSCLC is surgical excision; however, the prognosis remains poor (6). In addition, chemotherapy with or without surgery is not effective in the majority of cases; therefore, it is essential to identify novel compounds, including natural products, which may be employed for the treatment of lung cancer.

Cantharidin (CTD) is a component of mylabris (blister beetle), which has previously been used as a Traditional Chinese Medicine (7). Previous studies have reported that CTD induced cytotoxic effects in leukemia stem cells (8) as well as U937 (9), pancreatic cancer (10), hepatocellular carcinoma (11,12), colon cancer (13) and human lung cancer A549 (14) cells. In addition, CTD was found to inhibit the activity of protein phosphatase 2A (PP2A) (9) and heat shock factor 1 (HSF1) (15). Furthermore, it was shown that CTD induced cell death in human colorectal cancer cells, which was suggested to proceed through inhibiting the binding of heat shock protein 70 (HSP70), B cell lymphoma 2-associated athanogene domain 3 (BAG3) and HSF1 to promoters (15).

Genetic mutations in oncogenes and tumor suppressor genes are present in cancer cells (16,17). The development of cancer cells is well-known to be dependent on oncogenes for tumor initiation and progression; this concept has therefore been named oncogene addiction (18). Oncogenes are commonly used as targets for drug-screening programs (19); however, other signaling pathways have also been examined, such as the molecular chaperone pathway (20). The present study aimed to investigate the effect of CTD on the expression of key genes and functional pathways of human H460 lung cancer cells using complementary DNA microarray analysis. The results of the present study showed that CTD affected DNA damage, the cell cycle and the expression of apoptosis-associated genes *in vitro*. Differentially expressed genes were then used to generate interaction maps of signaling pathways. The epidermal growth factor and vascular endothelial growth factor receptor pathways, provided by the present study may be useful for the development of novel molecular targeted therapies against lung cancer (21).

## Materials and methods

### Chemicals and reagents

Cantharidin (CTD), propidium iodide and dimethyl sulfoxide (DMSO) were purchased from Sigma-Aldrich (St. Louis, MO, USA). Minimum essential medium (MEM), fetal bovine serum (FBS), L-glutamine and penicillin-streptomycin were purchased from Gibco-BRL (Carlsbad, CA, USA). CTD was dissolved in DMSO and stored at 20°C.

### Lung cancer cell culture

The NCI-H460 human lung cancer cell line was purchased from the Food Industry Research and Development Institute (Hsinchu, Taiwan). Cells were grown in MEM containing 10% (v/v) FBS as well as 100 U/ml penicillin and 100 *μ*g/ml streptomycin in a 37°C humidified incubator with 5% CO_2_. Cells were then subcultured once they reached 80–90% confluence, as previously described (22).

### Complementary (c)DNA microarray assay

H460 cells were placed on 12-well plates at a density of 5×10^5^ cells/well in 2 ml MEM with 10% (v/v) FBS and 2 mM L-glutamine, as well as 100 U/ml penicillin and 100 *μ*g/ml streptomycin for 24 h. Subsequently, cells were treated with or without 10 *μ*M CTD for a further 24 h. Cells (3×10^6^) were then harvested and washed twice with phosphate-buffered saline (Gibco-BRL). Cells were lysed in TRIzol^®^ (Invitrogen Life Technologies, Carlsbad, CA, USA) and total RNA was extracted using a Qiagen RNeasy Mini kit (Qiagen, Valencia, CA, USA). RNA concentrations were determined using a Qubit™ Fluorocytometer (Invitrogen Life Technologies).

Total RNA of CTD-treated and untreated H460 cells was used for cDNA synthesis. Samples were hybridized using an Affymetrix GeneChip Human Gene 1.0 ST array (Affymetrix, Santa Clara, CA, USA) according to the manufacturer’s instructions. Sample fluorescence was quantified by Asia BioInnovations Corp. (Taipei, Taiwan), while data were analyzed using the Transcriptome Analysis Console™ 2.0 Version 2.0.0.9. (Affymetrix) with default robust multichip analysis parameters. A 2-fold change in gene expression was used as the threshold to indicate an effect on expression (7–10). An Oligo(dT) Maxime RT PreMix kit (iNtRON Biotechnology, Gyeonggi-do, South Korea) was used to reverse transcribe RNA into cDNA. The Affymetrix GeneChip^®^ Whole Transcript Sense Target (ST) Labeling (cat. no. 900673; 30 Rxn; Affymetrix, Santa Clara, CA, USA) assay is designed to generate amplified and biotinylated sense-strand DNA targets from the entire expressed genome without bias. This assay and associated reagents have been optimized specifically for use with the GeneChip^®^ ST arrays, and the probes on the arrays have been selected to be distributed throughout the entire length of each transcript. The gene list complete with Affymetrix transcript identifiers, was uploaded from a spreadsheet onto Metacore 5.0 software (GeneGo pathways analysis; http://www.genego.com). GeneGo recognizes the Affymetrix identifiers and maps the gene to the MetaCore™ data analysis suite, generating maps to describe common pathways or molecular connections between genes in the list. Graphical representations of the molecular associations between the genes were generated using the GeneGo pathway analysis, based upon processes exhibiting a significant association (P<0.05).

### Gene ontology analysis

For detection of significantly over-represented GO biological processes, the DAVID functional annotation clustering tool (http://david.abcc.ncifcrf.gov) was used (DAVID Bioinformatics Resources 6.7). Enrichment was determined at the DAVID calculated Benjamini value <0.05. The significance of the overexpression of individual genes was determined using Student’s t-test.

### Statistical analysis

Values are representative of three independent experiments. Differences between control and CTD-experimental groups are presented which >2-fold, where positive numbers represent upregulation and negative numbers represent downregulation.

## Results

### Upregulated and downregulated gene expression in H460 cells exposed to CTD

H460 cells were incubated in the presence or absence of 10 *μ*M CTD in a 12-well plate for 24 h. Cells were then harvested and following the extraction of total RNA, RNA concentrations were determined and cDNA microarray analysis was performed in order to determine the expression of genes. The calculated upregulation and downregulation of gene expression, as determined by the microarray, are shown in Tables I and II, respectively. As shown in Table I, the results indicated that in CTD-treated H460 cells, 8 genes were upregulated >4-fold, 29 genes were upregulated >3–4-fold and 156 genes were upregulated >2–3-fold compared with expression levels in the untreated control cells. In addition, Table II indicated that one gene was downregulated >4 fold, 14 genes were downregulated >3–4 fold and 150 genes were downregulated >2–3 fold in H460 cells following exposure to CTD compared with those in the untreated control cells. The results presented in Table I demonstrated that genes associated with DNA damage, including DN1T3 and GADD45A were upregulated by 2.26-and 2.60-fold, respectively; in addition, the expression of genes associated with the cell cycle progression (check point proteins) were upregulated, including CCND2, CDKL3 and RASA4, which were upregulated 2.72-, 2.19- and 2.72-fold, respectively. Furthermore, the expression of apoptosis-associated genes was upregulated, such as CARD6, which was upregulated 3.54-fold (Table I). By contrast, the results presented in Table II demonstrated that genes associated with DNA damage, cell cycle progression and apoptosis were also downregulated, including DdiT4, CDC42EP3 and STAT2, respectively. These genes were found to be downregulated 3.14-, 2.16 and 2.04-fold, respectively (Table II). Overall, cDNA microarray analysis of H460 cells following treatment with CTD demonstrated that CTD induced the differential expression of numerous genes associated with DNA damage, cell cycle progression and apoptosis.

### GeneGo analysis

**Figure 1:**
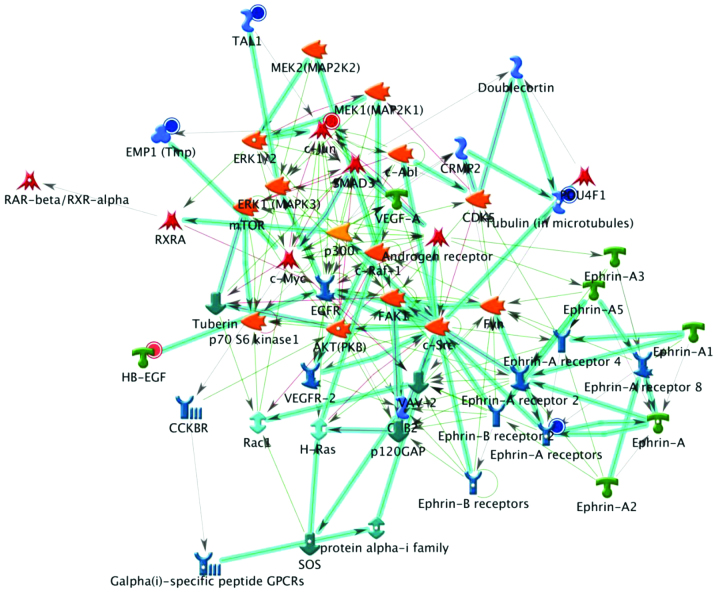
Top scored (by the number of pathways) AN network, as determined using GeneGo_cat_FC2 analysis. Thick cyan lines indicate the fragments of canonical pathways. Upregulated genes are marked with red circles and downregulated genes are indicated with blue circles. FC, Fold Change 2.0; AN, Analyze Networks algorithm.

A GeneGo analysis program was used to analyze the CTD-treated NCI-H460 cells in order to determine the top scoring genes which were differentially expressed, as determined by the number of pathway networks involved. The results of the GeneGo analyses are shown in Figs. 1^3^, which reveal the top, second and third scored genes by the number of pathways, respectively. Experimental data were used to generate maps of the pathway interactions and genes which were upregulated (indicated by red circles) and down-regulated (indicated by blue circles) in H460 cells following treatment with CTD. It was indicated that these genes may also be involved in DNA damage, cell cycle arrest and apop-tosis-associated responses in CTD-treated H460 cells.

## Discussion

CTD has been reported to have cytotoxic effects in numerous different types of cancer cell (8–15). The results of previous studies have also demonstrated that CTD-induced cell death occurred due to the induction of apoptosis in human lung cancer cells (data not shown) (23). However, the effects of CTD on gene expression in cancer cells have remained to be elucdiated. To the best of our knowledge, the present study was the first to report on the effects of CTD on gene expression in H460 cells. Therefore, the present study not only advanced the understanding of the differential gene expression following treatment with CTD in lung cancer cells, but may additionally provide several potential biomarkers for use as future therapeutic clinical targets for the treatment of lung cancer.

It has been well documented that the tumor microenvironment, which contains matrix proteins, stromal cells and associated secreted molecules, including cytokines and associated genes, which may be used as targets of cancer therapeutic drugs (24–26). Therefore, an increasing number of studies focus on elucidating the tumor microenvironment and associated gene expression in order to determine potential novel therapeutic agents for treating cancer patients (27). Over the past decade, there have been numerous clinical trials of treatments for lung cancer patients, including adjuvant chemotherapy trials and neo-adjuvant chemotherapy trials (28–30); however, the results of these trials have not yet provided a successful, effective treatment for lung cancer. Numerous studies have demonstrated that chemotherapeutics may result in cell death through DNA damage, cell cycle arrest and the induction of apoptosis (31,32). In the present study, H460 cells were treated with CTD and incubated in 12-well plates, and their RNA was then isolated in order to determine which genes exhibited altered expression following treatment with CTD. The results revealed that CTD effected the upregulation and downregulation, respectively, of the expression of certain genes which are known to be associated with DNA damage, cell cycle progression and apoptosis in H460 cells.

In order to further elucidate the molecular signaling pathways associated with altered gene expression in H460 cells following exposure to CTD, GeneGo Process Networks were used in the present study in order to analyze the altered gene expression results of the microarray, in order to determine the possible signaling pathways involved. Based on GeneGo pathway and canonical pathway maps, which represent a set of ~650 signaling and metabolic maps covering human biology (signaling and metabolism) in a comprehensive way. A preset network of protein interaction characteristics for the process was used for each process, and the experimental data were mapped regarding the specific process. The obtained hypothetical molecular signaling pathways indicated that CTD affects numerous associated signaling pathways, indicated by the involvement of the differentially expressed genes in the network of the respective the signaling pathways. The gene content of the uploaded files was used as the input list for the generation of biological networks using the Analyze Networks algorithm with default settings. This is a variant of the shortest paths algorithm, with main parameters of relative enrichment with the uploaded data, and relative saturation of the networks with canonical pathways. The network provides data listing interacting proteins. In this workflow the network is prioritized based on the number of fragments of canonical pathways on the network.

In conclusion, the results of the present study revealed that treatment with CTD induced the upregulation and downregulation of numerous genes in H460 cells. In addition, these differentially expressed genes were associated with DNA damage, cell cycle progression and apoptotic cell death in human lung cancer H460 cells. The present study also revealed possible signaling pathways, which may provide more information on the possible mechanism of CTD in H460 cells; however, further studies are required.

## Figures and Tables

**Figure 2:**
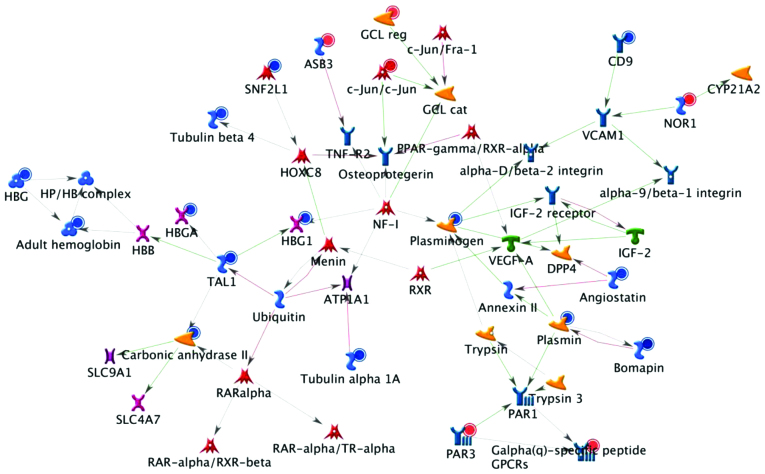
Second scored (by the number of pathways) AN network, as determined using GeneGo_cat_FC2 analysis. Thick cyan lines indicate the fragments of canonical pathways. Upregulated genes are marked with red circles and downregulated genes are indicated with blue circles. FC, Fold Change 2.0; AN, Analyze Networks algorithm.

**Figure 3:**
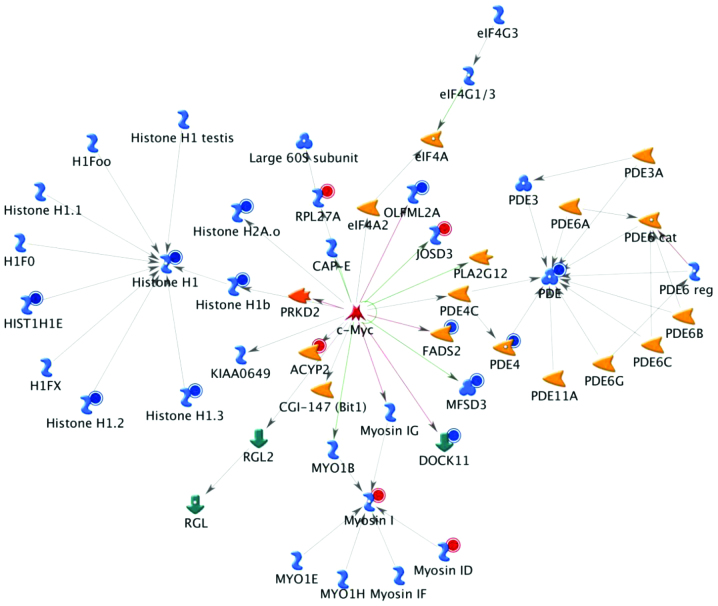
Third scored (by the number of pathways) AN network, as determined using GeneGo_cat_FC2 analysis. Thick cyan lines indicate the fragments of canonical pathways. Upregulated genes are marked with red circles and downregulated genes are indicated with blue circles. FC, Fold Change 2.0; AN, Analyze Networks algorithm.

**Table I:** Upregulation of gene expression in catharidine-treated NCI-H460 cells.

Probe set ID	Fold change	Gene symbol	Gene description	mRNA accession no.
8108370	16.50	EGR1	Early growth response 1	NM_001964
8012949	13.05	CDRT1	CMT1A duplicated region transcript 1	NM_006382
8012951	10.85	CDRT1	CMT1A duplicated region transcript 1	NM_006382
7916609	8.05	JUN	Jun oncogene	NM_002228
7977075	6.45	SNORA28	Small nucleolar RNA, H/ACA box 28	NR_002964
8041168	5.54	SNORD53	Small nucleolar RNA, C/D box 53	NR_002741
7982084	5.06	SNORD115-11	Small nucleolar RNA, C/D box 115-11	NR_003303
8097991	4.27	TDO2	Tryptophan 2,3-dioxygenase	NM_005651
8114468	3.99	SNORD63	Small nucleolar RNA, C/D box 63	NR_002913
8158862	3.87	SNORD62A	Small nucleolar RNA, C/D box 62A	NR_002914
8158864	3.87	SNORD62A	Small nucleolar RNA, C/D box 62A	NR_002914
8007420	3.85	AOC3	Amine oxidase, copper-containing 3 (vascular adhesion protein 1)	NM_003734
7975779	3.81	FOS	FBJ murine osteosarcoma viral oncogene homolog	NM_005252
8001746	3.74	SNORA46	Small nucleolar RNA, H/ACA box 46	NR_002978
7914322	3.70	SNORD103A	Small nucleolar RNA, C/D box 103A	NR_004054
7914324	3.70	SNORD103A	Small nucleolar RNA, C/D box 103A	NR_004054
793342	3.69	PTPN20A	Protein tyrosine phosphatase, non-receptor type 2	NR_001042389
7918467	3.65	Clorf103	Chromosome 1 open reading frame 103	NM_018372
8053797	3.60	LOC400986	Protein immuno-reactive with anti-parathyroid hormone polyclona	ENST00000456556
8156848	3.59	NR4A3	Nuclear receptor subfamily 4, group A, member 3	NM_006981
8005483	3.56	FBXW10	F-box and WD repeat domain-containing 10	NM_031456
8105077	3.54	CARD6	Caspase recruitment domain family, member 6	NM_032587
8139840	3.48	ERV3	Endogenous retroviral sequence 3 (includes zinc)	NM_001007253
8030831	3.45	ZNF175	Zinc finger protein 175	NM_007147
7958200	3.45	EID3	EP300-interacting inhibitor of differentiation 3	NM_001008394
7936637	3.44	SNORA19	Small nucleolar RNA, H/ACA box 19	NR_002917
8107353	3.43	ZRSR1	Zinc finger (CCCH type), RNA-binding motif and serine/arginine rich 1	BC104811
8173600	3.26	NAP1L2	Nucleosome assembly protein 1-like 2	NM_021963
8126853	3.25	C6orf138	Chromosome 6 open reading frame 138	NM_001013732
8025301	3.20	CD209	CD209 molecule	NM_021155
7923119	3.17	ZBTB41	Zinc finger and BTB domain-containing 41	NM_194314
7985555	3.11	EFTUD1	Elongation factor Tu guanine triphosphate binding domain-containing	NM_024580
7952986	3.09	HSN2	Hereditary sensory neuropathy, type II	NM_213655
8114572	3.08	HBEGF	Heparin-binding epidermal growth factor-like growth factor	NM_001945
8049540	3.05	LRRFIP1	Leucine-rich repeat (in FLII) interacting protein 1	NM_001137550
8047926	3.03	MAP2	Microtubule-associated protein 2	NM_002374
7954382	3.02	PYROXD1	Pyridine nucleotide-disulphide oxidoreductase domain 1	NM_024854
7957260	2.99	GLIPR1	GLI pathogenesis-related 1	NM_006851
8054054	2.97	ANKRD36B	Ankryin repeat domain 36B	NM_025190
7907572	2.96	PAPPA2	Pappalysin 2	NM_020318
8090688	2.93	SNORA58	Small nucleolar RNA, H/ACA box 58	NR_002985
8139935	2.89	TYW1B	tRNA-yW synthesizing protein 1 homolog B	NM_001145440
7982028	2.87	SNORD115-11	Small nucleolar RNA, C/D box 115-11	NR_003303
7982050	2.87	SNORD115-11	Small nucleolar RNA, C/D box 115-11	NR_003303
7982064	2.87	SNORD115-11	Small nucleolar RNA, C/D box 115-11	NR_003303
7982078	2.87	SNORD115-11	Small nucleolar RNA, C/D box 115-11	NR_003303
7982092	2.87	SNORD115-11	Small nucleolar RNA, C/D box 115-11	NR_003303
7905339	2.86	GABPB2	GA binding protein transcription factor, β subunit	NM_144618
8140782	2.84	ABCB1	ATP-binding cassette, sub-family B (multidrug resistance protein/transporter associated with antigen processing)	NM_000927
8139482	2.83	SNORA5A	Small nucleolar RNA, H/ACA box 5A	NR_002919
8124756	2.83	PPP1R10	Protein phosphatase 1, regulatory (inhibitor) subunit	NM_002714
8124756	2.83	PPP1R10	Protein phosphatase 1, regulatory (inhibitor) subunit	NM_002714
8178358	2.83	PPP1R10	Protein phosphatase 1, regulatory (inhibitor) subunit	NM_002714
8179664	2.83	PPP1R10	Protein phosphatase 1, regulatory (inhibitor) subunit	NM_002714
8112731	2.77	F2RL2	Coagulation factor II (thrombin) receptor-like 2	NM_004101
8043687	2.74	ANKRD36	Ankryin repeat domain 36	NM_001164315
7977273	2.74	ADSSL1	Adenylosuccinate synthase like 1	NM_152328
7953200	2.72	CCND2	Cyclin D2	NM_001759
8057954	2.72	C2prf66	Chromosome 2 open reading frame 66	AY358249
8049532	2.72	LRRFIP1	Leucine-rich repeat (in FLII) interacting protein 1	NM_001137550
8141843	2.72	RASA4	RAS p21 protein activator 4	NM_006989
8098752	2.72	ABCA11P	Adenosine triphosphate-binding cassette, sub-family A, member 11, pseudogene	NR_002451
7982046	2.70	SNORD115-20	Small nucleolar RNA, C/D box 115-20	NR_003312
7982016	2.70	SNORD115-12	Small nucleolar RNA, C/D box 115-12	NR_003304
7982024	2.70	SNORD115-12	Small nucleolar RNA, C/D box 115-12	NR_003304
7982030	2.70	SNORD115-12	Small nucleolar RNA, C/D box 115-12	NR_003304
8054064	2.69	ANKRD36B	Ankyrin repeat domain 36B	NM_025190
7910047	2.68	DNAH14	Dynein, axonemal, heavy chain 14	NM_001373
8016239	2.68	PLEKHM1	Pleckstrin homology domain-containing, family M (with RUN domain) member 1	NR_027774
8060949	2.67	ANKRD5	Ankyrin repeat domain 5	NM_022096
8064375	2.62	SRXN1	Sulfiredoxin 1 homolog (*S. Cerevisiae*)	NM_080725
8077612	2.61	TTLL3	Tubulin tyrosine ligase-like family, member 3	NM_001025930
8151559	2.60	SLC10A5	Solute carrier family 10 (sodium/bile acid cotransporter family), member 5	NM_001010893
7902227	2.60	GADD45A	Growth arrest and DNA-damage-inducible, α	NM_001924
7982058	2.59	SNORD115-26	Small nucleolar RNA, C/D box 115-26	NR_003343
8118023	2.55	GTF2H4	General transcription factor II human, polypeptide 4	NM_001517
8006336	2.54	LRRC37B	Leucine-rich repeat-containing 37B	NM_052888
8108006	2.51	LEAP2	Liver-expressed antimicrobial peptide 2	NM_052971
7971388	2.50	SLC25A30	Solute carrier family 25, member 30	NM_001010875
7987163	2.48	FMN1	Formin 1	ENST00000414268
7938293	2.47	SNORA45	Small nucleolar RNA, H/ACA box 45	NR_002977
8113651	2.45	ATG12	ATG12 autophagy-related 12 homolog (*S. Cerevisiae*)	NR_033362
8049542	2.45	LRRFIP1	Leucine-rich repeat (in FLII) interacting protein 1	NM_001137550
8097435	2.45	C4orf33	Chromosome 4 open reading frame 33	NM_173487
8168345	2.45	ACRC	Acidic repeat-containing	NM_052957
7980828	2.42	CCDC88C	Coiled-coil domain-containing 88C	NM_001080414
8045423	2.42	SNORA40	Small nucleolar RNA, H/ACA box 40	NR_002973
8112331	2.42	ISCA1	Iron-sulfur cluster assembly 1 homolog	NM_030940
8043697	2.41	ANKRD36B	Ankyrin repeat domain 36B	NM_025190
8033667	2.40	ZNF558	Zinc finger protein 558	NM_144693
8142232	2.39	LAMB4	Laminin, β4	NM_007356
7960052	2.39	SNORA49	Small nuclear RNA, H/ACA box 49	NR_002979
8031837	2.39	ZNF587	Zinc finger protein 587	AF294842
8174715	2.38	SNORA69	Small nuclear RNA, H/ACA box 69	NR_002584
8001748	2.38	SNORA50	Small nuclear RNA, H/ACA box 50	NR_002980
8042503	2.38	MXD1	MAX dimerization protein 1	NM_002357
8072678	2.36	HMOX1	Heme oxygenase (decycling) 1	NM_002133
7997904	2.35	ZNF778	Zinc finger protein 778	AK295122
8053648	2.35	KRCC1	Lysine-rich coiled-coil 1	NM_016618
8035793	2.35	ZNF737	Zinc finger protein 737	NM_001159293
7977732	2.34	SNORD8	Small nuclear RNA, C/D box 8	NR_002916
8153457	2.31	EEF1D	Eukaryotic translation elongation factor 1δ	AY358690
8069574	2.31	C21orf91	Chromosome 21 open reading frame 91	NM_001100420
8112841	2.30	HOMER1	Homer homolog 1 (*Drosophila*)	NM_004272
8038919	2.29	ZNF350	Zinc finger protein 50	NM_021632
9175288	2.29	MOSPD1	Motile sperm domain-containing 1	NM_019556
8160912	2.28	C9orf131	Chromosome 9 open reading frame 131	NM_203299
7934334	2.28	TTC18	Tetratricopeptide repeat domain 18	NM_145170
8056572	2.27	SPC25	SPC25, NDC80 kinetochore complex component	NM_020675
8161919	2.27	TLE1	Transducin-like enhancer of split 1 (*Drosophila*)	NM_005077
7964460	2.26	DDIT3	DNA-damage-inducible transcript 3	NM_004083
8019857	2.26	NDC80	NDC80 Homolog, kinetochore complex component (*S. cerevisiae*)	NM_006101
8045587	2.24	ACVR2A	Activin A receptor, type IIA	NM_001616
8002660	2.24	TXNL4B	Thioredoxin-like 4B	NM_017853
7911329	2.24	14-Sep	Septin 14	NM_207366
8080980	2.24	FLJ10213	Endogenous Borna-like N element-1	NM_018029
8014115	2.22	MYOID	Myosin ID	NM_015194
7949916	2.20	CHKA	Choline kinase α	NM_001277
7938295	2.20	RPL27A	Ribosomal protein L27a	NM_000990
8168146	2.20	KIF4A	Kinesin family member 4A	NM_012310
8114171	2.19	CDKL3	Cycline-dependent kinase-like 3	NM_001113575
8008700	2.19	FLJ11710	Hypothetical protein FLJ11710	AK021772
8108321	2.18	FAM53C	Family with sequence similarity 53, member C	NM_001135647
8047161	2.18	OBFC2A	Oligonucleotide/oligosaccharide-binding fold-containing 2a	NM_001031716
8053576	2.17	RNF103	Ring finger protein 103	NM_005667
8006237	2.17	LOC400590	Hypothetical LOC400590	ENST00000433145
8136341	2.17	BPGM	2,3-bisphosphoglycerate mutase	NM_199186
8146225	2.16	C8orf40	Chromosome 8 open reading frame 40	NM_001135674
8147057	2.16	CHMP4C	Chromatin modifying protein 4C	NM_152284
7921228	2.15	ETV3	E26 transforming-specific variant 3	NM_001145312
8065607	2.15	PLAGL2	Pleiomorphic adenoma gene-like 2	NM_002657
8096511	2.14	BMPR1B	Bone morphogenetic protein receptor, type 1B	NM_001203
7927389	2.14	MAPK8	Mitogen-activated protein kinase 8	NM_002750
8098958	2.14	POLN	Polymerase (DNA directed) nu	NM_181808
8038989	2.14	ZNF600	Zinc finger protein 600	NM_198457
7951654	2.14	FDXACB1	Ferredoxin-fold anticodon binding domain-containing 1	NM_138378
8036341	2.13	ZNF461	Zinc finger protein 461	NM_153257
7981998	2.13	SNORD116-25	Small nucleolar RNA, C/D box 116-25	NM_003339
8041179	2.13	CLIP4	Cytoskeleton-associated protein-glycine-rich domain-containing linker protein family member 4	NM_024692
7969096	2.13	CDADC1	Cytidine and deoxycytidine monophosphate deaminase domain-containing 1	NM_030911
7969243	2.13	CKAP2	Cytoskeleton-associated protein 2	NM_018204
7986350	2.12	ARRDC4	Arrestin domain-containing 4	NM_183376
8063382	2.12	SNAI1	Snail homolog 1 (*Drosophila*)	NM_005985
8053801	2.12	ANKRD36	Ankyrin repeat domain 36	NM_001164315
7999588	2.12	PLA2G10	Phospholipase A2, group X	NM_003561
8008795	2.11	C17orf71	Chromosome 17 open reading frame 71	NM_018149
8029340	2.11	ZNF155	Zinc finger protein 155	NM_003445
8166104	2.11	OFD1	Oral-facial-digital syndrome 1	NM_003611
8123825	2.11	SLC35B3	Solute carrier family 35 member B3	NM_015948
7901052	2.11	SNORD38B	Small nucleolar RNA, C/D box 38B	NM_001457
8084880	2.10	HES1	Hairy and enhancer of split 1	NM_005524
7925672	2.10	ZNF670	Zinc finger protein 670	NM_033213
7982294	2.10	OTUD7A	OTU domain-containing 7A	NM_130901
7962112	2.09	CAPRIN2	Caprin family member 2	NM_001002259
7973948	2.09	BRMSIL	Breast cancer metastasis-suppressor 1-like	NM_032352
8117685	2.09	ZKSCAN3	Zinc finger with KRAB and SCAN domains 3	NM_024493
8010082	2.08	SNORD1A	Small nucleolar RNA, C/D box 1A	NR_004395
8041982	2.08	ACYP2	Acylphosphatase 2, muscle type	NM_138448
8137693	2.08	COX19	Cytochrome c oxidase assembly homolog 19	NM_001031617
7917779	2.08	GCLM	Glutamate-cysteine ligase, modifier subunit	NM_002061
7938364	2.08	WEE1	WEE1 homolog 1 (*S. Pombe*)	BX641032
8007414	2.08	AOC2	Amine oxidase, copper-containing 2 (retina-specific)	NM_009590
8139656	2.08	GRB10	Growth factor receptor-bound protein 10	NM_001001555
8059852	2.08	MSL3L2/MSL3-like 2	Male-specific lethal 3-like 2 (*Drosophila*)	NM_001166217
8109484	2.07	KIF4B	Kinesin family member 4B	NM_001099293
8022559	2.07	ANKRD29	Ankyrin repeat domain 29	NM_173505
7910030	2.07	DNAH14	Dynein, axonemal, heavy chain 14	NM_00145154
8052143	2.07	GPR75	G protein-coupled receptor 75	NM_006794
7931643	2.07	CYP2E1	Cytochrome P450, family 2, subfamily E, polypeptide 1	NM_000773
8102789	2.06	TERF1	Telomeric repeat binding factor (NIMA-interacting) 1	NM_003218
7953603	2.06	C1S	Complement component 1, s subcomponent	NM_201442
8104930	2.05	SLC1A3	Solute carrier family 1 (glial high affinity glutamate transporter), member 3	NM_004172
7953211	2.05	C12orf5	Chromosome 12 open reading frame 5	NM_020375
8114326	2.04	FAM13B	Family with sequence similarity 13, member B	NM_0166603
7936826	2.04	IKZF5	IKAROS family zinc finger (Pegasus)	NM_022466
8013567	2.04	C17orf108	Chromosome 17 open reading frame 108	NM_001076680
7975066	2.04	AKAP5	A-kinase anchor protein 5	NM_004857
8142524	2.04	TSPAN12	Tetraspanin 12	NM_012338
7952673	2.04	FLJ45950	FLJ45950 protein	AK127847
8081128	2.04	NSUN3	Nucleolar protein 2 homolog/Sun domain family, member 3	NM_022072
7922846	2.04	FAM129A	Family with sequence similarity 129, member A	NM_052966
8013305	2.04	ZNF286B	Zinc finger protein 286B	NM_001145045
8153935	2.03	ZNF252	Zinc finger protein 252	NM_023392
8162490	2.03	HIATL1	Hippocampus abundant transcript-like 1	NM_032558
8128698	2.02	SESN1	Sestrin 1	NM_014454
8010778	2.02	CSNK1D	Casein kinase 1, δ	NM_001893
8141311	2.02	FAM200A	Family with sequence similarity 200, member A	NM_145111
7944867	2.02	SIAE	Sialic acid acetylesterase	NM_170601
7961829	2.02	BCAT1	Branched-chain amino-acid transaminase 1, cytosolic	NM_005504
7994161	2.02	RBBP6	Etinoblastoma binding protein 6	NM_006910
7981273	2.02	CCDC85C	Coiled-coil domain-containing 85C	NM_001144995
8110649	2.02	TRIM41	Tripartite motif-containing 41	NM_033549
8101839	2.01	EIF4E	Eukaryotic translation initiation factor 4E	NM_001968
8103226	2.01	TMEM154	Transmembrane protein 154	NM_152680

**Table II:** Downregulation of gene expressions in catharidine-treated NCI-H460 cells.

Probe set ID	Fold change	Gene symbol	Gene description	mRNA accession no.
8175016	−3.08	APLN	Apelin	NM_017413
8124413	−3.06	HIST1H4D	Histone cluster 1, H4d	NM_003539
8105302	−3.05	FST	Follistatin	NM_006350
7953665	−3.04	DPPA3	Developmental pluripotency-associated 3	NM_199286
8117426	−2.99	HIST1H2BH	Histone cluster 1, H2bh	NM_003524
8117898	−2.97	HIST1H4J	Histone cluster 1, H4j	NM_021968
8117337	−2.95	HIST1H1E	Histone cluster 1, H1e	NM_005321
7911241	−2.93	OR2L8	Olfactory receptor, family 2, subfamily L, member 8	NM_001001963
8048749	−2.88	KCNE4	Potassium voltage-gated channel, IsK-related family, member 4	NM_080671
8124437	−2.87	HIST1H3F	Histone cluster 1, H3f	NM_021018
8117395	−2.83	HIST1H2BF	Histone cluster 1, H2bf	NM_003522
8015798	−2.79	LOC100130581	Hypothetical LOC100130581	NR_027413
7919642	−2.78	HIST2H2AB	Histone cluster 2, H2ab	NM_175065
8059470	−2.76	IRS1	Insulin receptor substrate 1	NM_005544
8077270	−2.75	CHL1	Cell adhesion molecule with homology to L1 cell adhesion molecule	NM_006614
7915592	−2.74	RNU5D	RNA, U5D small nuclear	NR_002755
7906767	−2.74	FCGR2C	Fc fragment of immunoglobulin G, low affinity IIc, receptor for (CD32) (gene/pseudogene)	NM_201563
8117594	−2.74	HIST1H2BM	Histone cluster 1, H2bm	NM_003521
8117589	−2.72	HIST1H3H	Histone cluster 1, H3h	NM_003536
7981728	−2.71	LOC100293211	Similar to hCG2042717	ENST00000390601
8124397	−2.71	HIST1H1C	Histone cluster 1, H1c	NM_005319
8135734	−2.71	C7orf58	Chromosome 7 open reading frame 58	NM_024913
8138988	−2.70	DPY19L2P1	Dpy-19-like 2 pseudogene 1 (*C. elegans*)	NR_002833
8117583	−2.65	HIST1H2AI	Histone cluster 1, H2ai	NM_003509
8153258	−2.65	SLC45A4	Solute carrier family 45, member 4	BC033223
7960865	−2.61	SLC2A3	Solute carrier family 2 (facilitated glucose transporter), member 3	NM_006931
8116921	−2.59	EDN1	Endothelin 1	NM_001955
8101757	−2.58	GPRIN3	G protein-regulated inducer of neurite outgrowth family member 3	NM_198281
7971723	−2.58	FLJ37307	Hypothetical LOC283521	NR_027047
8117580	−2.56	HIST1H2AI	Histone cluster 1, H2ai	NM_003509
8167573	−2.56	GAGE1	G antigen 1	NM_001468
8165295	−2.56	LCN8	Lipocalin 8	ENST00000371686
7927876	−2.53	TET1	Ten-eleven translocation oncogene 1	NM_030625
7963054	−2.52	TUBA1A	Tubulin, α1a	NM_006009
7957386	−2.51	ACSS3	Acyl-CoA synthetase short-chain family member 3	NM_024560
7915919	−2.49	TAL1	T cell acute lymphocytic leukemia 1	NM_003189
8174985	−2.48	SMARCA1	Switch/sucrose non-fermentable-related, matrix-associated, actin-dependent regulator of chromatin, subfamily a, member 1	NM_003069
8105495	−2.47	PART1	Prostate androgen-regulated transcript 1 (non-protein coding)	NR_028508
8146967	−2.46	CRISPLD1	Cysteine-rich secretory protein LCCL domain-containing 1	NM_031461
8144786	−2.46	SLC7A2	Solute carrier family 7 (cationic amino acid transporter, y+ system), member 2	NM_003046
8029280	−2.45	CD177	CD177 molecule	NM_020406
7984524	−2.45	PAQR5	Progestin and adipoQ receptor family member V	NM_001104554
7973182	−2.44	LOC554207	Hypothetical LOC554207	ENST00000320322
8145795	−2.44	LOC100293539	Similar to ribosomal protein 10	XM_002346094
8095697	−2.44	CXCL1	Chemokine (C-X-C motif) ligand 1 (melanoma growth stimulating activity, α)	NM_001511
8124406	−2.42	HIST1H2BC	Histone cluster 1, H2bc	NM_003526
7978260	−2.42	DHRS1	Dehydrogenase/reductase family member 1	NM_001136050
7920271	−2.42	S100A4	S100 calcium binding protein A4	NM_019544
8166469	−2.42	SAT1	Spermidine/spermine N1-acetyltransferase 1	NR_027783
8124527	−2.40	HIST1H1B	Histone cluster 1, H1b	NM_005322
8008321	−2.40	ACSF2	Acyl-CoA synthetase family member 2	NM_025149
8124385	−2.39	HIST1H4B	Histone cluster 1, H4b	NM_003544
800731	−2.38	TUBG2	Tubulin, γ2	NM_016437
8122365	−2.36	GPR126	G protein-coupled receptor 126	NM_020455
8015273	−2.34	KRT31	Keratin 31	NM_002277
7904465	−2.34	HIST2H2BA	Histone cluster 2, H2ba	NR_027337
8124416	−2.33	HIST1H3D	Histone cluster 1, H3d	NM_003530
8074458	−2.33	C22orf39	Chromosome 22 open reading frame 39	NM_173793
8046048	−2.33	CSRNP3	Cysteine-serine-rich nuclear protein 3	NM_001172173
8007493	−2.32	ARL4D	Adenosine diphosphate-ribosylation factor-like 4D	NM_001661
8157804	−2.31	OLFML2A	Olfactomedin-like 2A	NM_182487
7948836	−2.31	TMEM223	Transmembrane protein 223	NM_001080501
8033458	−2.31	LYPLA2	Lysophospholipase II	NM_007260
7919619	−2.30	HIST2H2AA3	histone cluster 2, H2aa3	NM_003516
7905079	−2.30	HIST2H2AA3	Histone cluster 2, H2aa3	NM_003516
7927631	−2.29	DKK1	Dickkopf homolog 1 (*Xenopus laevis*)	NM_012242
7975598	−2.28	ACOT1	Acyl-CoA thioesterase 1	NM_001037161
8071801	−2.27	GSTTP1	Glutathione S-transferase θ pseudogene 1	NR_003081
7928429	−2.27	PLAU	Plasminogen activator, urokinase	NM_002658
8068898	−2.27	HIST1H2BK	Histone cluster 1, H2bk	NM_080593
8041467	−2.26	VIT	Vitrin	NM_053276
8077160	−2.26	ARSA	Arylsulfatase A	NM_000487
7991754	−2.25	HBZ	Hemoglobin, ζ	NM_005332
8049534	−2.24	LRRFIP1	Leucine-rich repeat (in FLII) interacting protein 1	NM_001137550
7909789	−2.23	TGFB2	Transforming growth factor β2	NM_001135599
7919612	−2.23	HIST2H3D	Histone cluster 2, H3d	NM_001123375
8100578	−2.22	EPHA5	Ephrin receptor A5	NM_004439
8169541	−2.22	DOCK11	Dedicator of cytokinesis 11	NM_144658
8124430	−2.21	HIST1H1D	Histone cluster 1, H1d	NM_005320
8124524	−2.21	HIST1H2AK	Histone cluster 1, H2ak	NM_003510
8124524	−2.21	HIST1H2AK	Histone cluster 1, H2ak	NM_003510
7906775	−2.20	HSPA7	Heat shock 70kDa protein 7 (HSP70B)	NR_024151
7953291	−2.20	CD9	CD9 molecule	NM_001769
8033319	−2.19	SH2D3A	Src Homology 2 domain-containing 3A	NM_005490
7905088	−2.19	HIST2H2AC	Histone cluster 2, H2ac	NM_003517
7975602	−2.19	ACOT2	Acyl-CoA thioesterase 2	NM_006821
7982854	−2.19	DLL4	δ-like 4 (*Drosophila*)	NM_019074
8019778	−2.19	PCYT2	Phosphate cytidylyltransferase 2, ethanolamine	NM_002861
8046048	−2.19	HIST1H4C	Histone cluster 1, H4c	NM_003542
8007493	−2.18	VWA5A	Von Willebrand factor A domain-containing 5A	NM_001130142
8157804	−2.18	PLG	Plasminogen	NM_000301
7948836	−2.18	CD24	CD24 molecule	NM_013230
8033458	−2.17	FSTL4	Follistatin-like 4	NM_015082
7919619	−2.16	CA2	Carbonic anhydrase II	NM_000067
7905079	−2.16	CDH19	Cadherin 19, type 2	NM_021153
7927631	−2.16	CDC42EP3	Cell division cycle 42 effector protein (Rho guanosine triphosphatase binding) 3	NM_006449
7975598	−2.16	ACCN2	Amiloride-sensitive cation channel 2, neuronal	NM_020039
8071801	−2.15	HIST2H3D	Histone cluster 2, H3d	NM_001123375
7928429	−2.15	RFX2	Regulatory factor X, 2 (influences human leukocyte antigen class II expression)	NM_000635
8068898	−2.15	NES	Nestin	NM_006617
8041467	−2.15	LOC25845	Hypothetical LOC25845	NR_024158
8077160	−2.15	THSD7A	Thrombospondin, type I, domain-containing 7A	NM_015204
7991754	−2.14	LOC147727	Hypothetical LOC147727	NR_024333
8049534	−2.14	CALML6	Calmodulin-like 6	NM_138705
7909789	−2.14	DEFB109P1B	Defensin, β 109, pseudogene 1B	NR_003668
7919612	−2.13	EPOR	Erythropoietin receptor	NM_000121
8100578	−2.13	EEF2K	Eukaryotic elongation factor-2 kinase	NM_013302
8169541	−2.13	EMP3	Epithelial membrane protein 3	NM_001425
8124430	−2.13	TMEM84	Transmembrane protein 84	NR_026949
8124524	−2.13	CXXC5	CXXC finger 5	NM_016463
7906775	−2.12	PCYT2	Phosphate cytidylyltransferase 2, ethanolamine	NM_002861
7953291	−2.12	LYPD1	Ly6/plasminogen activator, urokinase 1 receptor domain-containing	NM_144586
8033319	−2.12	PHLDB2	Pleckstrin homology-like domain, family B, member 2	NM_001134439
7905088	−2.11	LRFN2	Leucine-rich repeat and fibronectin type III domain-containing 2	NM_020737
7975602	−2.11	C9orf23	Chromosome 9 open reading frame 23	NM_148179
7982854	−2.11	FLJ13744	Hypothetical FLJ13744	BC070061
8018445	−2.11	UNK	Unkempt homolog (*Drosophila*)	NM_001080419
8038407	−2.10	RRAS	Related RAS viral (r-ras) oncogene homolog	NM_006270
7987230	−2.10	LPCAT4	Lysophosphatidylcholine acyltransferase 4	NM_153613
8031514	−2.10	LOC100133142	Hypothetical LOC100133142	XM_001718400
8130374	−2.10	FBXO5	F-box protein 5	NM_012177
7908409	−2.09	RGS2	Regulator of G-protein signaling 2	NM_002923
8111255	−2.09	CDH10	Cadherin 10, type 2 (T2-cadherin)	NM_006727
7965335	−2.09	DUSP6	Dual specificity phosphatase 6	NM_001946
8065537	−2.09	LOC100134868	Hypothetical LOC100134868	NR_004846
8138466	−2.08	MACC1	Metastasis associated in colon cancer 1	NM_182762
7902687	−2.08	CYR61	Cysteine-rich, angiogenic inducer, 61	NM_001554
8036136	−2.08	TMEM149	Transmembrane protein 149	NM_024660
8098916	−2.08	TMEM129	Transmembrane protein 129	NM_001127266
7955663	−2.07	TENC1	Tensin-like C1 domain-containing phosphatase (tensin 2)	NM_170754
7939897	−2.07	FOLH1	Folate hydrolase (prostate-specific membrane antigen) 1	NM_004476
7920191	−2.07	LCE3A	Late cornified envelope 3A	NM_178431
7951437	−2.06	GUCY1A2	Guanylate cyclase 1, soluble, α2	NM_000855
8022653	−2.06	LOC728606	Hypothetical LOC728606	NR_024259
7929816	−2.06	SCD	Stearoyl-CoA desaturase (δ-9-desaturase)	NM_005063
7940565	−2.06	FADS2	Fatty acid desaturase 2	NM_004265
7951157	−2.06	CCDC82	Coiled-coil domain-containing 82	AK313893
7936100	−2.06	CALHM2	Calcium homeostasis modulator 2	NM_015916
7954090	−2.06	EMP1	Epithelial membrane protein 1	NM_001423
8005951	−2.05	SNORD42B	Small nucleolar RNA, C/D box 42B	NR_000013
8148917	−2.05	MFSD3	Major facilitator superfamily domain-containing 3	NM_138431
7937990	−2.04	HBG1	Hemoglobin, γA	NM_000559
7937993	−2.04	HBG2	Hemoglobin, γG	NM_000184
8033233	−2.04	TUBB4	Tubulin, β4	NM_006087
8048350	−2.04	PLCD4	Phospholipase C, δ4	NM_032726
8037408	−2.04	KCNN4	Potassium intermediate/small conductance calcium-activated channel, subfamily N, member 4	NM_002250
7964119	−2.04	STAT2	Signal transducer and activator of transcription 2	NM_005419
8016841	−2.03	TMEM100	Transmembrane protein 100	NM_001099640
7958948	−2.03	DDX54	DEAD box polypeptide 54	NM_0011111322
8151512	−2.02	PAG1	Phosphoprotein associated with glycosphingolipid microdomains 1	NM_018440
8005549	−2.02	GRAPL	Growth factor receptor-bound protein 2-related adaptor protein-like	NM_001129778
8033159	−2.02	PSPN	Persephin	NM_004158
7986639	−2.02	VSIG6	V-set and immunoglobulin domain-containing 6	ENST00000338567
7938741	−2.01	MRGPRX3	MAS-related G protein coupled receptor, member X3	NM_054031
8047174	−2.01	SLC39A10	Solute carrier family 39 (zinc transporter), member 10	NM_001127257

Acyl-CoA, acyl coenzyme A; DEAD, (Asp-Glu-Ala-Asp).
